# A Comparison of the Effect of Sevoflurane and Propofol on Ventricular Repolarisation after Preoperative Cefuroxime Infusion

**DOI:** 10.1155/2019/8978906

**Published:** 2019-01-02

**Authors:** Yanqiu Liu, Hong Gao, Guilong Wang, Li An, Jing Yi, Xiaokui Fu, Chunlei Wen, Zijun Wang

**Affiliations:** ^1^Department of Anesthesiology, The Affiliated Hospital of Guizhou Medical University, Guiyang, Guizhou, China; ^2^Department of Anesthesiology, Guizhou Medical University, Guiyang, Guizhou, China

## Abstract

The aim of this study is to investigate the changes in QT, QTc, and Tp-e intervals and Tp-e/QT ratio on surface electrocardiogram (ECG) signals during anaesthesia induction using propofol or sevoflurane after preoperative cefuroxime infusion. Some 120 cases of gynaecological patients are randomly divided into propofol (P) and sevoflurane (S) groups (n=60). After cefuroxime (1.5 g) was infused in the two groups of patients, propofol target controlled infusion (TCI) was conducted in the P group for 5 min to realise a plasma concentration of 4 *μ*g/ml while patients in the S group inhaled anaesthesia by infusing 1.3 MAC sevoflurane for 6 min. The 12-lead ECGs were separately collected before infusing cefuroxime (T_1_), after infusing cefuroxime (T_2_), and after infusing propofol or sevoflurane (T_3_) to measure QT and Tp-e intervals, calculate QTc and Tp-e/QT, and record MAP and HR. Finally, we demonstrated that QT, QTc, and Tp-e intervals and Tp-e/QT ratio had no change (*P* > 0.05) after cefuroxime infusion in the two groups of patients compared with that before infusing antibiotics. Moreover, after conducting preoperative cefuroxime infusion, using propofol and sevoflurane had no influence on Tp-e interval, but sevoflurane can significantly prolong QT and QTc intervals (*P* < 0.05).

## 1. Introduction

Antibiotics are the favourite drugs used to prevent and cure infectious diseases; however, the cardiotoxicity caused by antibiotics cannot be ignored with the extending application in clinical practice [1–3]. It is reported that antibiotics can prolong QT and QTc intervals [4–6]. Prolongation of the QT interval increases transmural dispersion of repolarisation (TDR) of the ventricle, probably increasing the risk of arrhythmia risk [5]. A case report indicates that antibiotics can lead to torsade de pointes [7]. Generally, cephalosporin antibiotics are infused in surgical patients preoperatively to prevent postoperative wound infection [8, 9]. In intraoperative periods, anaesthetic drugs also influence QT and QTc intervals [10–14]. Moreover, propofol (intravenous anaesthetic) probably shortens, prolongs, or does not change the QTc interval [10–12, 14] while sevoflurane (inhalation anaesthetic) prolongs the QT interval [14]. Antibiotics and anaesthetics both have effects on QT interval. It has not been clarified whether using propofol or sevoflurane, after antibiotic infusion, will aggravate abnormal ventricular repolarisation and increase TDR so as to increase perioperative arrhythmia. The Tp-e interval, the period from the peak-to-the end of the T-wave, is a remarkable reflection of TDR in ECG and can flexibly predict risk of arrhythmogenesis. In contrast, the ratio of Tp-e/QT excludes the influence of HR [14–16]. The study investigated the changes of QT, QTc, and Tp-e intervals and the ratio of Tp-e/QT in ECG during anaesthesia induction by using propofol or sevoflurane after infusing cefuroxime. By doing so, the study evaluated the safety of patients after being anaesthetised using propofol or sevoflurane in view of cardiac electrophysiology, which provides references for future clinical anaesthesia.

## 2. Methods

The experiment has been reviewed and approved by Medical Ethics Committee in the college (Application Number: 20150124, Guizhou Medical University), and the subjects have given informed consent prior to participating in the study.

### 2.1. Study Populations and Design

There were 120 cases of elective gynaecological laparoscopic operation (hysteromyomectomy, excision of ovarian cyst, and excision of ovarian tumors) during January to November 2016 and patients aged from 20 to 50 showed ASA I or II. The patients were found to have normal preoperative cardiopulmonary function, ECG, and electrolyte state, and the HR-corrected QT (QTc) interval was less than 440 ms. They did not take medication for prolonging the QT interval including antiarrhythmic drugs, blocking agent for *β* receptors, antidepressants, or phenothiazine drugs. Moreover, they did not have diabetes or other endocrine diseases. The patients were randomly divided into propofol (P) and sevoflurane (S) groups using a random number table, with 60 cases in each group.

### 2.2. Procedure

Preoperative fasting and water-deprivation of patients separately last for 8 h and 4 h and no patient took any preoperative medication. To prevent the influence of diurnal change on QT interval, all of the experimental data were collected in the morning (8:30 to 11:30 A.M.) [17]. After entering the operating room, the routine monitoring of MAP, HR, and SpO_2_ of patients was conducted. After opening the upper extremity vein (UEV), 130/0.4 sodium chloride injection (10 mL/kg) of hydroxyethyl starch (specification: 500 mL/bag, batch number: 81KC081, Beijing Fresenius Kabi Medical Group) was infused to the UEV within 30 min. The ECG electrode was established and the first ECG was obtained by using the ECG-1250C 12-lead electrocardiograph (Shanghai Photoelectric Medical Instrument Corporation) at a chart speed of 25 mm/s and gain amplification of 10 mm/mV. Afterwards, cefuroxime sodium (antibiotic) (1.5 g: batch number: 505316, specification: 0.75 g each, Esseti Farmaceutici Srl, Italy) was put into 100 mL normal saline and then intravenously infused within 20 min to collect the second ECG. Next, the propofol (diprivan, 10 mg/mL, batch number: LC699; Corden Pharma S.P.A., Italy) TCI (Graseby Anaesthesia Pump 3500, UK) was applied to patients in the P group. The third ECG was collected after the plasma concentration reached the targeted level of 4 *μ*g/mL and was sustained for 5 min. Inhalation induction was conducted to anaesthetise patients in the S group using sevoflurane (batch number: 64181, specification: 250 mL/bottle, Maruishi Pharmaceutical Co., Ltd, Japan). Before induction, 8%v/v of sevoflurane and oxygen flow at 6 L/min was applied to prefill the breathing circuit and the oxygen flow was adjusted to 2 L/min after the patients fell asleep. Moreover, the concentration in the volatilisation pot was adjusted and the third ECG was collected after the sevoflurane concentration reached 1.3 MAC and was sustained for 6 min. During the research period, patients maintained autonomous respiration and then they breathed via an oxygen mask after using the anaesthetic (assisted ventilation if necessary). During the period, SpO_2_ and P_ET_CO_2_ were maintained at 99% to 100% and 35 to 40 mmHg, respectively.

The QT, QTc, and Tp-e intervals and the ratio of Tp-e/QT of the two groups of patients before infusing antibiotics (T_1_), after infusing antibiotics (T_2_), and after propofol TCI or sevoflurane inhalation for specific time (T_3_) were separately measured. Moreover, MAR and HR at T_1_ to T_3_ were recorded.

### 2.3. Electrocardiographic Measurements

The ECG data were analysed according to uniform standards after completing the observation. According to literature [18] the V4 lead was selected to measure the QT and Tp-e intervals. The QT interval was measured from the Q-wave start to the T-wave end while the Tp-e interval was measured from the peak-to-the end of the T-wave. Here, the peak of T wave refers to the peak point of the T-wave and the terminal point of the T-wave represents the intersection point between the declined branch tangent of the T-wave and the base line. If a U-wave appears, the terminal point of the T-wave is the incisura between the T- and U-waves [14]. Three continuous, complete QT and Tp-e intervals were measured by using the V4 lead to calculate the average value [13, 19]. According to the Bazett formula (QTc = QT/$\sqrt{RR}$), the QTc and the ratio of Tp-e/QT were calculated [10].

### 2.4. Statistical Analysis

Statistical analysis was conducted by using SPSS 19.0 software. The measurement data are represented by using mean and standard deviation $\left(\overset{-}{x}\pm s\right)$. Comparisons within group were conducted through repeated measures analysis of variance and a* t*-test was employed for comparison between groups:* P *< 0.05 denotes a statistically significant difference.

## 3. Results

(1) By comparing the two groups of patients under general conditions, it can be seen that difference is not statistically significant (*P *> 0.05), as shown in Table 1.

(2) By comparing the MAP and HR of the two groups of patients at different time points, the difference was not statistically significant (*P* > 0.05), as shown in Table 2.

(3) The comparison of QT, QTc, and Tp-e intervals and the ratio of Tp-e/QT of the two groups of patients at different times is summarised in Table 3. The QT, QTc, and Tp-e intervals and the ratio of Tp-e/QT of the two groups of patients at T_1_ and T_2_ were compared and the difference was insignificant (*P *> 0.05). Compared with T_1_ and T_2_, the QT and QTc intervals of the S group were prolonged at T3 (*P *< 0.05) while the ratio of Tp-e/QT decreased (*P *< 0.05). Compared with the P group, the QT and QTc intervals of the S group were prolonged at T3 (*P *< 0.05) while the ratio of Tp-e/QT decreased (*P *< 0.05).

## 4. Discussion

Sudden cardiac death (SCD) occurs in about 800,000 people every year, abnormal ventricular repolarisation-induced malignant centricular arrhythmia (MVA) is the most common reason for SCD in clinical practice [20]. Numerous drugs can trigger MVA including antibiotics [5]. Milberg et al. [21] suggested that all antibiotics can trigger abnormal ventricular repolarisation and prolong the QT interval [21]. In 2010, the American College of Cardiology Foundation and The American Heart Association (ACCF/AHA) indicated that the risk of* torsades de pointes* (TdP) significantly increases if the QTc interval exceeds 500 ms [22]. The mechanisms of antibiotic-induced cardiotoxicity are manifested in the following two ways: on the one hand, antibiotics block the rapid delayed rectifier potassium (I_kr_) channel of cardiomyocytes; on the other hand, there is a pharmacokinetic effect between antibiotics and other drugs [23].

Preoperative antibiotic infusion can prevent postoperative infection [1–3]. Cephalosporin antibiotics are the most commonly used in clinical practice [6, 8]. Moreover, as anaesthetic also influences the QT interval, the safety aspects of using anaesthetics after antibiotic infusion in clinical practice are worth noting.

The QT, QTc, and Tp-e intervals and the ratio of Tp-e/QT in ECG are indices reflecting the ventricular repolarisation and can predict the risk of arrhythmia to some extent [14, 16]. The QT interval refers to the duration from the initiation of the QRS complex to the termination of the T-wave, representing the whole process of ventricular depolarisation and repolarisation [14]. Easily affected by HR, QT is generally represented by using a corrected QT (QTc) interval [14]. QT or QTc interval prolongation is frequently used to evaluate the risk of endogenous TdP with various drugs [5, 14]. When depolarisation is prolonged (QRS ≥120 ms), the QT interval is also prolonged so that a single prolongation of repolarisation time cannot cause arrhythmia [24]. The transmural reentrant is considered as the primary mechanism of arrhythmia while TDR increase is regarded as an electrophysiological basis for multiple ventricular arrhythmias [22, 25, 26]. TDR refers to the electrical heterogeneity during the repolarisation of epicardium, endocardium, and midmyocardium, which is shown as the Tp-e interval in a surface ECG [14, 16]. As a quantitative index of TDR, the Tp-e interval exerts an important guiding significance on predictions of ventricular arrhythmia and evaluation of the risk therein. The conduction of excitation can be influenced when the Tp-e interval is prolonged, causing reentry and even inducing TdP [25–27]. The Tp-e interval is normally in the range of 85 ± 11 ms with an upper limit of 110 ms in white and Hispanic women [28]. The Tp-e interval is also affected by HR while the ratio of Tp-e/QT maintains unchanged within the normal range of 0.15 to 0.25 [15, 16]. The Tp-e interval and the ratio of Tp-e/QT are new indices able to evaluate and predict ventricular arrhythmias [18].

The QT interval is a dynamic physiological variable. The probability of QT interval prolongation increases in populations of more than 65, or less than 18 years of age, and females are more likely to suffer from QT interval prolongation than males [29, 30]. So, young, middle-aged females are chosen for observation. As multiple factors (e.g., autonomic nerve activity, electrolyte concentration of plasma, and haemodynamic stability) also influence the QT interval [29, 30], preoperative electrolyte levels in all test subjects were verified as normal and no preoperative drugs were used. The haemodynamics were maintained in a stable state during the research to reduce interference effects on the observed indices.

A TCI for targeted 4 *μ*g/mL of plasma concentration was used in the P group. The target plasma concentration rose to target effect-site concentration after 3 to 5 min, so the observation time-point was set at 5 min after reaching the target plasma concentration of propofol (T_2_). The 1.3 MAC sevoflurane was inhaled by patients in the S group. The time constant of brain tissue with sevoflurane is 2 min and partial pressures of brain tissue and pulmonary alveoli are balanced after three times that time constant, so the time point was set at 6 min after sevoflurane reached 1.3 MAC (T_2_) [17, 19, 31].

The research results show that QT, QTc, and Tp-e intervals and the ratio of Tp-e/QT were unchanged after cefuroxime infusion, implying that the prophylaxis infusion of single-dose cefuroxime of 1,500 mg has no effect on ventricular repolarisation. Chen et al. showed that the QTc interval is significantly prolonged after healthy volunteers take 400 mg of moxifloxacin [4] and Kazuhiro et al. suggested that the QT interval can increase after 10 mg/kg of ciprofloxacin is infused in guinea pigs by intravenous injection [6]. In spite of this, Shampa et al. found that single-dose prophylaxis infusion of 2,000 or 3,000 mg of ceftaroline and 2,000 mg of avibactam in healthy women does not influence the QT interval [8]. Moreover, Riccobene et al. also revealed that single-dose prophylaxis infusion of 1,500 mg of ceftaroline in healthy women does not influence the QTc interval [6]. In the research, single-dose cefuroxime was first used for patients, which possibly did not show influences on ventricular repolarisation: when infusing cefuroxime for a long time, or repeatedly, the influence of cefuroxime on ventricular repolarisation may well be observed.

By using the anaesthesia induction of propofol TCI after infusing cefuroxime, the QT, QTc, and Tp-e intervals and the ratio of Tp-e/QT remained unchanged (*P *> 0.05). This indicates that using propofol as anaesthesia induction after cefuroxime infusion cannot influence ventricular repolarisation and disturb the stability of cardiac electrophysiology. The result is similar to that obtained by Hume-Smith et al. on children; that is, propofol TCI at plasma concentrations of 3 to 6 *μ*g/mL has no effect on QT and Tp-e intervals in healthy children [29]. The influence of propofol on ventricular repolarisation is still a matter of debate [10–12]. Kim et al. revealed that the QT interval of patients significantly prolongs after propofol (5 *μ*g/mL) TCI induction [12]. Higashijima et al. point out that the QT interval is shortened after applying 1.5 mg/kg propofol to maintain anaesthesia in patients undergoing spinal surgical procedures [11]; however, Bolat et al. reported that anaesthesia using 30 mg of propofol exerted no significant influence on the QT interval of patients undergoing upper abdominal endoscopy [10].

By using sevoflurane inhalation induction after infusing cefuroxime, QT and QTc intervals were significantly extended (*P* < 0.05). The mechanism is probably related to constraint of the slowly activated delayed rectifier potassium current (Iks) by using sevoflurane [16, 19, 32]; however, the Tp-e interval remained unchanged and the ratio of Tp-e/QT decreased (*P *< 0.05), which implies that although 1.3 MAC sevoflurane can extend the duration of myocardiac repolarisation, it does not increase the nonuniformity thereof. The result is consistent with the conclusion obtained by Lee et al. to the effect that 8% of sevoflurane used for anaesthesia induction of children only prolongs QTc interval but does not influence TDR [16]. Owing to sevoflurane significantly extending the QT interval, it is necessary to prevent the risk of TdP when children with congenital LQTS are anaesthetised using sevoflurane [5].

The study shows certain shortcomings: firstly, only female patients with normal preoperative QT intervals were studied. Considering the fact that females have a longer QT interval, they are more easily influenced by drugs used to prolong the interval. Therefore, the research result is probably not suitable for application among male patients. Secondly, after infusing cefuroxime, propofol with 4 *μ*g/mL of target controlled concentration of plasma, and sevoflurane with 1.3 MAC was used; however, the influence of propofol with different doses or sevoflurane with different concentrations on ventricular repolarisation after infusing cefuroxime was not further explored. Thirdly, the safety of using anaesthesia during operations in patients with long QT intervals or having other antibiotic infusions before operations remains to be further studied.

In summary, using propofol and sevoflurane after preoperative cefuroxime infusion show no influence on TDR; however, sevoflurane significantly prolongs ventricular repolarisation duration. Thus, it is necessary to prevent the risk of arrhythmia in patients with prolonging QT interval or while using higher concentrations of sevoflurane before operations.

## Figures and Tables

**Table 1:** A comparison of the two groups of patients under general conditions (mean ± SD).

Group	Cases	ASA grade (i.e. I/II)	Age (Years)	Body weight (kg)
P group	60	42/18	37±8	56±7
S group	60	50/10	37±8	55±7

Data are presented as mean ± standard deviation.

**Table 2:** A comparison of MAP and HR of the two groups of patients at different times (mean ± SD).

Group	Cases	Index	T_1_	T_2_	T_3_
P group	60	MAP (mmHg)	76±9	76±7	73±9
		HR (beats/min)	73±9	75±9	73±10
S group	60	MAP (mmHg)	76±7	76±7	74±8
		HR (beats/min)	74±8	76±9	74±6

Data are presented as mean ± standard deviation.

(MAP= Mean arterial pressure, HR=Heart rate)

**Table 3:** A comparison of QT, QTc, and Tp-e intervals and the ratio of Tp-e/QT of the two groups at different times (mean ± SD).

Group	Cases	Index	T_1_	T_2_	T_3_
P group	60	QT (ms)	393.10±19.82	394.43±16.99	394.17±20.80
		QTc (ms)	419.25±12.43	422.25±14.04	424.55±17.25
		Tp-e (ms)	79.35±9.32	79.82±8.03	80.90±11.27
		Tp-e/QT	0.20±0.03	0.20±0.02	0.21±0.03
S group	60	QT (ms)	393.57±19.61	392.20±21.05	427.17±25.96^abc^
		QTc (ms)	418.95±10.97	421.05±11.69	442.43±11.89^abc^
		Tp-e (ms)	79.57±7.79	80.05±7.91	77.87±8.11
		Tp-e/QT	0.21±0.02	0.21±0.02	0.19±0.02^abc^

Data are presented as mean ± standard deviation.

Compared with T_1_, ^a^*P* < 0.05; compared with T_2_, ^b^*P* < 0.05; compared with the P group, ^c^*P* < 0.05.

## Data Availability

The data used to support the findings of this study are available from the corresponding author upon request.
